# Identifying Factors Associated with the Acquisition of Multiple Indications for Anticancer Drugs

**DOI:** 10.3390/curroncol33060339

**Published:** 2026-06-06

**Authors:** Shutaro Takahashi, Hideki Maeda

**Affiliations:** 1 Regulatory Science, Graduate School of Pharmaceutical Science, Meiji Pharmaceutical University, 2-522-1, Noshio, Kiyose-shi, Tokyo 204-8588, Japan; 2 Portfolio and Project Management, Oncology Development, Astellas Pharma Inc., 2-5-1 Nihonbashi-Honcho Chuo-ku, Tokyo 103-8411, Japan; d236753@std.my-pharma.ac.jp

**Keywords:** oncology, anticancer drugs, multiple indications, clinical efficacy, tumor type, company size, mechanism of action

## Abstract

With the declining success rate of new drug development, lifecycle management strategies have become increasingly important. However, approximately 25–40% of approved drugs remain limited to a single indication. In this study, we compared drugs that obtained multiple indications with those confined to a single indication, aiming to identify factors associated with the acquisition of multiple indications. We identified anticancer drugs initially approved by US Food and Drug administration (FDA) between 2015 and 2020. This retrospective observational study includes 48 anticancer drugs for solid tumors. For each drug, data were collected on approved indications, sponsoring company characteristics, and clinical efficacy outcomes, including objective response rate (ORR) and progression-free survival (PFS). The drugs were categorized based on the number of approved indications, and comparative analyses were conducted. No clear association was observed between multiple indication acquisition and factors such as mechanism of action (MoA) or company size at the initial approval. Since additional indications may be obtained through various approaches—including expansion to new tumor types or different lines of therapy—no consistent relationship was observed between specific strategies and the acquisition of multiple indications. In contrast, drugs demonstrating higher clinical efficacy and those initially approved for non-rare cancers were more likely to achieve multiple indications. The factors associated with additional indications varied depending on the approach, and only a limited set of factors consistently correlated with multiple indication acquisition. Notably, clinical efficacy and the tumor type at initial approval appeared to be associated with acquisition of multiple indications. These findings provide important insights into research and development strategies and may inform decision-making in pharmaceutical lifecycle management.

## Simple Summary

Declining drug-development success rates and rising R&D costs underscore the importance of effective lifecycle management, yet many anticancer drugs remain limited to a single indication. This study examined factors associated with multiple indication acquisition among solid tumor agents approved by the US FDA between 2015 and 2020. Data were collected on approved indications, mechanism of action of drugs, company characteristics, regulatory pathways, and clinical efficacy. No clear association was found between multiple indication acquisition and mechanism of action, company size or regulatory pathways. In contrast, drugs with higher clinical efficacy and those initially approved for non-rare cancers were more likely to gain additional indications. These findings suggest that clinical efficacy and initial tumor type are key determinants of indication expansion and may help inform development strategies and evidence-based decision-making in oncology lifecycle management.

## 1. Introduction

Novel drug development requires multiple stages, including basic pharmaceutical research, preclinical studies, clinical trials, and regulatory review. Consequently, the probability that an initially synthesized ultimately reaches regulatory approval is estimated to be as low as 1 in 20,000–30,000 [1,2]. Furthermore, research and development (R&D) costs have been continuously increasing [3,4], and the current development cost for a single new molecular entity is >1 billion USD [5]. Therefore, life-cycle management (LCM) strategies that maximize the therapeutic potential and commercial opportunity of an approved drug are important for sustaining pharmaceutical R&D [6,7]. An increasing number of drugs have obtained approvals for multiple indications in recent years. In this study, multiple indications were defined as two or more distinct approved indications listed in the regulatory label, including expansions by tumor type, line of therapy, biomarker-defined subgroup, stage or combination regimen.

Many drugs obtain multiple indications; approximately 60–75% of marketed medications have more than one indication [5,8]. In the present study, the most frequent type of label expansion involves a change in cancer type, followed by line of therapy, combination therapy, biomarker-based stratification, and disease stage [5]. Pembrolizumab (Keytruda) is a representative example of a drug that has repeatedly been filed for label expansion. The approved indications for pembrolizumab exceed 10 different cancer types. Although lung cancer represents the largest patient population among its indications, only approximately 30% of Keytruda’s sales in 2022 were derived from drugs indicated for lung cancer, whereas approximately 70% came from drugs indicated for other cancer types [9]. Although Pembrolizumab is not intended to represent all muti-indication drugs, it illustrates how repeated indication expansion can substantially broaden both clinical use and commercial value. This demonstrates the importance of indication expansion in realizing the full therapeutic and commercial potential of a drug. Moreover, indication expansion can enhance cost-efficiency in promotion through a “halo effect,” whereby brand recognition extends across multiple therapeutic areas [10]. Thus, pursuing additional indications represents an effective approach for companies to generate cash flow efficiently.

In typical development strategies, drug candidates are first tested in patients with advanced-stage cancers or in later lines of therapy and are subsequently expanded to earlier-stage cancers or first-line settings [11]. By selecting a small-population target indication, companies can increase the likelihood of regulatory approval even with less mature data, while also enhancing the possibility of obtaining favorable pricing based on the initial niche population. Furthermore, drug development for diseases with high unmet medical needs can take advantage of expedited review processes by regulatory authorities, which also yields high returns for pharmaceutical companies [12,13,14]. Previous research on multi-indication drugs has primarily focused on these development and pricing strategies [15,16,17]. However, 30–40% of approved drugs remain limited to a single indication. Despite the recognized importance of indication expansion, a substantial proportion of approved drugs remain limited to a single indication. Moreover, limited evidence is available on how drugs that remain confined to a single indication differ from those that subsequently obtain multiple indications. In this study, we compared drugs with only one approved indication to those with multiple indications. Specifically, we aimed to analyze their mechanism of actions (MoAs), sponsor company size, initially approved indication, and other development-related factors.

A “narrow first” strategy, initiating development in small-population indications, is common for multi-indication drugs [18,19]. This approach provides advantages such as high pricing potential [20,21], short time to initial approval [22,23], and a low regulatory hurdle due to the unmet medical need and relatively limited competition in such areas. We hypothesized that, although drugs with only a single indication are often developed in small-population indications with relatively low approval hurdles, subsequent expansion is abandoned due to limited overall drug potential. Because this study was exploratory, this hypothesis was used to guide descriptive comparisons rather than to establish causal relationships. We examined the cancer type of the lead indication and efficacy in clinical studies through comparison of single-indication drugs with those that obtained multiple indications. Furthermore, as label expansion requires considerable development investment, we also assessed the impact of the sponsoring company’s financial capacity.

Because FDA approval records and labels provide publicly accessible and standardized information on initial approvals, approved indications, and subsequent label expansions, FDA-approved drugs represent a suitable data source for systematically evaluating factors associated with indication expansion. The objective of this exploratory study was to identify factors associated with the acquisition of multiple indications among anticancer drugs initially approved by FDA between 2015 and 2020. We hypothesized that drugs remaining limited to a single indication may often achieve initial approval in populations with relatively limited development activity and less competition, but may face challenges in obtaining additional indications compared with drugs that demonstrate success in more actively developed therapeutic targets. Clarifying differences between single-indication and multi-indication drugs provide exploratory insights into R&D strategies for indication expansion.

## 2. Materials and Methods

### 2.1. Methods for Identifying Anticancer Drugs

We listed 831 drugs approved between 2015 and 2020 using the US Food and Drug Administration (FDA) database [24].

The search criterion was “Original NDA and Original BLA approval by months.” (NDA and BLA refer to New Drug Application and Biologics License Application respectively). The study period was limited to 2015–2020 to ensure sufficient follow-up for subsequent indication expansions while reducing temporal heterogeneity in development and regulatory practices.

For each drug name, we retrieved approval status and indications from the same database and extracted only anticancer drugs indicated for solid tumors. Only drugs with new active ingredients were included, and products containing the same active ingredient were excluded to avoid duplicate counting. In this study, antitumor drugs were manually defined as drug with at least one FDA-approved indication for the treatment of solid tumors. The number of approved indications was counted using the latest available FDA package insert as of December 2024.

Screening and data extraction were conducted by one reviewer. Ambiguous cases were discussed among the authors and resolved by consensus.

### 2.2. Clinical Data for Each Drug

For the remaining drugs, clinical trial information was obtained from ClinicalTrials.gov [25].

We extracted efficacy data such as ORR from the earliest conducted Phase 1 or 1/2 (First in human trial) and Phase 3 or 2/3 trials and median PFS from the earliest conducted Phase 3 or 2/3 trials. ORR and PFS were selected as descriptive efficacy measures because they are commonly reported in oncology trials and are relatively consistently extractable across drugs and development stages. Efficacy signals from FIH studies may influence subsequent development strategies and prioritization in pharmaceutical companies. Therefore, evaluating whether preliminary efficacy signals observed in FIH studies are associated with subsequent indication expansion is meaningful in an exploratory context. We also considered efficacy signals from pivotal trials as potentially informative indicators of successful lifecycle management.

When multiple cohorts were available, efficacy data with the largest sample size was prioritized. In a few cases where only aggregate dose-escalation data were reported, the aggregate value was extracted.

For trial results that were not reported in ClinicalTrials.gov, we searched PubMed using the trial identification number and extracted the relevant data.

The MoA of each drug was determined using the National Cancer Institute drug dictionary [26].

Overall survival was not included as a primary efficacy variable because OS was not consistently reported in early-phase trials and may be confounded by subsequent therapies, crossover, and differences in follow-up duration. In addition, because some drugs were approved based on single-arm trials or surrogate endpoints, OS data were sometimes unavailable or immature at the time of initial approval. Therefore, ORR and PFS were selected as more consistently extractable efficacy measures across development stages.

### 2.3. Labeling Information

Drug indication details were obtained from the prescribing information available in the FDA database. These details included tumor type, monotherapy or combination therapy status, cancer stage, treatment line, perioperative or maintenance therapy information, and biomarker requirements. For expanded indications, only updated prescribing information reflecting changes in the approved indication was used. Because this study focused on solid tumors, for drugs approved for both solid tumors and hematologic malignancies, only indications for solid tumors were counted. Of the 48 included drugs, 5 had approved indications for both solid tumors and hematologic malignancies.

### 2.4. Sponsoring Companies

Sponsoring companies were identified from the FDA database.

Company size was determined based on financial reports for the fiscal year preceding approval, and companies were classified as Emerging Biopharma (annual revenue < 500 million USD and R&D < 200 million USD), Large Pharma (revenue ≥ 10 billion USD), or Other Pharma, which did not meet either definition [27]. The study was conducted in accordance with the Strengthening the Reporting of Observational Studies in Epidemiology (STROBE) guidelines for cross-sectional observational studies [28].

## 3. Results and Discussion

### 3.1. Background Characteristics of Approved Drugs

The FDA approved 831 drugs between 2015 and 2020. Of these, 75 were cancer-related drugs. Biosimilars, drugs indicated only for hematologic malignancies, and those with non-anticancer therapeutic effects such as adverse event management were subsequently excluded. In total, 48 drugs met the criteria for inclusion in this study (Figure S1). Table 1 presents the background characteristics of these drugs. In total, 20 drugs had only one indication, 14 drugs had two indications, and 14 drugs had three or more indications. Approximately 40% of drugs approved between 2015 and 2020 remained limited to a single indication.

### 3.2. Impact of the Cancer Type at Initial Approval

**Figure 1:**
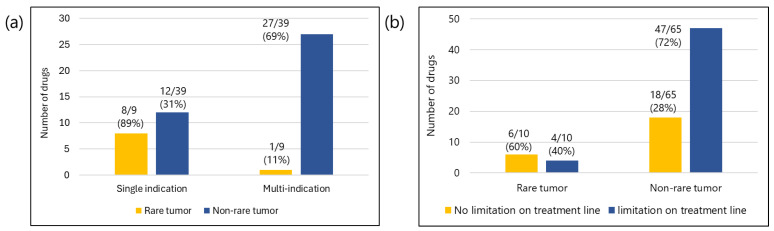
Association between tumor type and approval conditions at initial approval and acquisition of multiple indications. Numbers above bars indicate n/N (%). N represents the total number of drugs in each tumor-type group. (**a**) Relationship between tumor rarity at initial approval and the number of indications obtained. (**b**) Association between tumor rarity and treatment-line-related approval conditions across approved indications.

We first investigated potential characteristic trends related to the type of cancer at the time of initial approval. 8 single-indication drugs were initially approved for rare cancers, whereas only one (11%) multi-indication drug was approved for a rare cancer. In contrast, among drugs first approved for non-rare cancers, 12 remained limited to a single indication, whereas 27 (69%) acquired multiple indications (Figure 1). Rare cancers were identified according to the Rare Cancer Classification of National Cancer Institute [29], which is based on the RARECARE classification defined rare cancers with an annual crude incidence rate of fewer than 6 per 100,000 population. According to this definition, gastrointestinal stromal tumors, biliary tract cancer, sarcomas, Merkel cell carcinoma, neuroblastoma, and tenosynovial giant cell tumors were classified as rare cancers.

In oncology, specific drugs are generally recommended for use at defined lines of therapy. For example, enfortumab vedotin was initially approved for locally advanced/metastatic urothelial carcinoma (la/mUC) in patients previously treated with both PD-1/PD-L1 inhibitors and platinum-based chemotherapy [30]. Subsequently, its indication was expanded to include treatment-naïve patients with la/mUC in combination with pembrolizumab [31,32]. Thus, obtaining approval in a restricted treatment line may serve as a gateway for future label expansion [33]. Accordingly, we examined the approval conditions with respect to treatment line for all approved indications, including both initial and additional indication, stratified by rare versus non-rare cancers (Figure 1). Among rare cancers, 60% of drugs had no specification regarding treatment line, compared with 28% of drugs for non-rare cancers.

These findings suggest that regulatory approval may be granted without restrictions on treatment line in rare cancers, where patient populations are small and few approved therapies are available. In the case of Ayvakit and Tazverik [34,35], patient populations are defined by biomarkers rather than by treatment line. In contrast, drugs such as Turalio and Lenvima have obtained approvals for rare cancers without imposing restrictions such as biomarker status or treatment line [36,37]. Such broader initial approval setting in rare cancers may partly explain why subsequent label expansion to multiple indications is more challenging for these drugs.

Using biomarkers instead of treatment line to define the patient population and demonstrate novelty is not limited to rare cancers. This strategy can also be applied to major tumor types such as non-small cell lung cancer, as exemplified by Retevmo and Mektovi [38,39].

### 3.3. Impact of MoA on Indication Expansion

Our analysis further revealed that the most common form of indication expansion involves the addition of new cancer types (Figure S2) [40,41]. While initiating development in rare cancers generally precludes strategies that broaden treatment lines, expansion into additional cancer types can provide opportunities to increase market share. Therefore, we investigated the relationship between a drug’s MoA and its pattern of indication expansion (Figure S3a).

When the patterns of cancer-type expansion were examined by MoA, molecular targeted inhibitors tended to remain approved within a single cancer type, whereas monoclonal antibodies, including immune checkpoint inhibitors, were more frequently approved across multiple cancer types. This observation may reflect the fact that some antibody-based agents target molecular or immune pathways shared across different malignancies [42,43]. Although tumor-agnostic development has been established for selected molecular targeted inhibitors in biomarker-defined populations, such as NTRK fusion- or RET fusion-positive tumors, these cases represent specific biomarker-driven strategies rather than a generalizable feature of all molecular targeted inhibitors. In our dataset, molecular targeted inhibitors were more frequently approved within a single cancer type, indicating that tumor-agnostic development could not be generalized to the molecular targeted inhibitors analyzed in this study. No substantial differences were observed when analyzing the distribution of drugs with single versus multiple indications within each MoA category. Antibody–drug conjugates uniformly obtained approvals for multiple indications, although their sample size was small and consequently warrants cautious interpretation. Overall, we found no conclusive evidence that MoA alone strongly affects the likelihood of obtaining multiple indications (Figure S3b).

Addition of combination therapy to existing monotherapy is a common approach for indication expansion. In recent years, combination therapy development has increased compared with single-agent development [44]. As combination strategies are generally driven by efforts to enhance efficacy, overcome resistance mechanisms and toxicity management [45,46,47], MoA may also serve as a relevant factor influencing the development of combination therapies. For molecular targeted drugs, a manageable toxicity profile may support the feasibility of combination regimen development, although it is not the primary rationale for combination strategies. Although MoA may influence the direction of indication expansion, the data suggests that various other factors also play a role. Thus, no definitive relationship was established in this study.

### 3.4. Impact of Drug Efficacy

**Figure 2:**
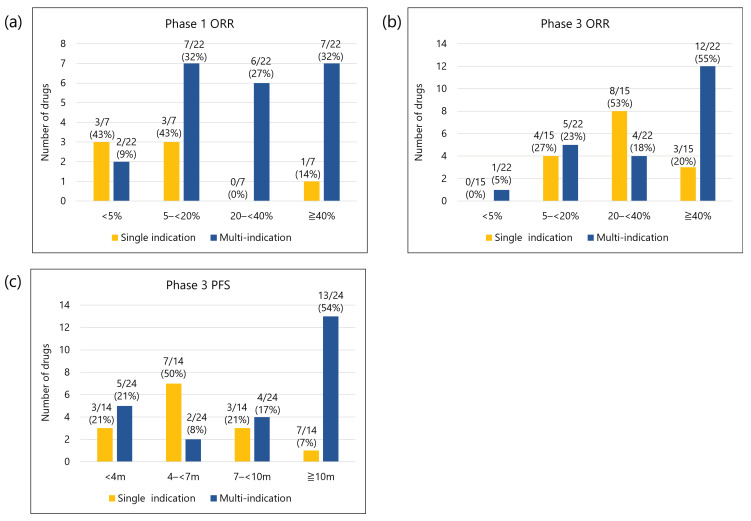
Association between clinical efficacy and the number of indications obtained. Numbers above bars indicate n/N (%). N represents the total number of drugs with available efficacy data in each indication group. (**a**) Phase 1 objective response rate (ORR) and number of indications; (**b**) Phase 3 ORR; (**c**) Phase 3 progression-free survival (PFS).

We further evaluated “clinical efficacy indications” and examined efficacy results from Phase 1 and 3 trials. Phase 1 ORRs are summarized in Figure 2a. Excluding 19 drugs for which data were unavailable, 29 drugs were included in the analysis. When the Phase 1 ORR was <5%, more than half of the drugs remained limited to a single indication. The proportion of drugs obtaining multiple indications also increased with increasing ORR. When the Phase 1 ORR exceeded 20%, only one of 7 drugs remained limited to a single indication.

A similar analysis was conducted for Phase 3 trials (Figure 2b). Results for 37 drugs were analyzed after excluding 11 drugs without reported efficacy data. Consistent with Phase 1 findings, a higher ORR was associated with a higher likelihood of acquiring multiple indications. In particular, when the ORR exceeded 40%, 12 out of 22 drugs obtained approval for multiple indications.

We also examined PFS in this study. Longer PFS was associated with a higher probability of multiple indication approvals. When PFS was 10 months or longer, the proportion of drugs with multiple indications was high. These findings suggest that drugs with ORRs > 40% or PFS longer than 10 months are likely to obtain multiple indications [48]. Conversely, drugs with an ORR below 20% or PFS < 7 months tended to remain limited to a single indication, potentially due to insufficient clinical potential, failure in further development, or prioritization of resources to other drugs with higher potential.

As shown in Table 1, clinical efficacy data were unavailable for some drugs in this study. Missing Phase 1 and Phase 3 efficacy data may have introduced selection and reporting biases. Early-phase efficacy data may be preferentially available for trials with favorable results, potentially leading to overestimation of early clinical activity. In addition, the absence of Phase 3 data may reflect different development pathways, particularly for drugs approved in rare cancers or high-unmet-need populations based on single-arm or early-phase studies. Thus, comparisons based on available efficacy data may partly reflect differences in data availability, target tumors, and regulatory strategy rather than true differences in drug efficacy. These findings should therefore be interpreted as exploratory.

### 3.5. Impact of Company Size

Companies with greater financial capacity often conduct multiple indication trials in parallel to rapidly expand market share. Conversely, smaller companies may face difficulties in developing drugs with multiple indications in mind [49]. Therefore, we investigated whether company size is associated with the acquisition of multiple indications (Figure S4). Among drugs developed by emerging biopharma companies, four had a single indication, whereas five had multiple indications. Among drugs developed by large pharmaceutical companies, nine had a single indication and 15 had multiple indications. However, no clear pattern was identified, suggesting that company size was not clearly associated with the acquisition of multiple indications.

### 3.6. Sample Representativeness and Limitations

In this study, we analyzed factors associated with the acquisition of multiple indications based on approval conditions and clinical trial data for drugs first approved by the FDA between 2015 and 2020. In total, 48 anticancer agents for solid tumors were included. The study period was intentionally restricted to a relatively short time span to minimize potential temporal confounding in identifying factors potentially associated with multi-indication approvals. Consequently, this work employed a narrative, qualitative and exploratory descriptive analytical approach based on a limited sample size, and no statistical analyses were performed. Therefore, the observed differences should be interpreted as descriptive trends. Future studies using larger datasets, longer follow-up periods are needed to validate and extend these exploratory findings. In addition, drugs initially approved in 2015 had a longer observation period and therefore more opportunity to obtain additional indications than drugs approved in 2020. As a result, drugs approved earlier in the study period may have been more likely to be classified as multi-indication drugs simply because they had more time for label expansion. Conversely, drugs approved later in the study period may still acquire additional indications in the future and may have been misclassified as single-indication drugs at the censoring date. Therefore, the observed differences between single-indication and multi-indication drugs may partly reflect differences in follow-up time rather than intrinsic differences in drug characteristics or development potential. Future studies with longer and uniform follow-up periods, or analyses accounting for time from initial approval to subsequent indication expansion, are warranted.

Another limitation of this study is that the analysis was restricted to drugs approved by the FDA. Approvals by other major regulatory authorities, such as the EMA, MHRA, and PMDA, were not included. Because regulatory frameworks may differ across authorities, the findings of this study may not be directly generalizable to other authorities. In addition, drugs that obtained additional indications outside the United States may have been misclassified as single-indication drugs in this FDA-based analysis. Therefore, future studies using datasets from multiple regulatory authorities are warranted to validate these findings and strengthen their external validity.

Several agents included in the analysis had indications for both solid and hematologic malignancies. However, because the analysis focused exclusively on solid tumors, the findings and conclusions may not be generalizable to drugs also approved for hematologic cancers. For drugs initially approved for hematologic malignancies and later approved for solid tumors, the first solid tumor indication was treated as the initial indication in this analysis; this applied only to TIBSOVO. Conversely, among drugs initially approved for solid tumors, only 4 agents—AYVAKIT, PEMAZYRE, TAZVERIK and COTELLIC—subsequently obtained indications for hematologic malignancies. Although these cases were limited in number, excluding hematologic malignancies may have introduced classification bias, because drugs with indications spanning both solid and hematologic malignancies could have been treated as single-indication drugs.

Although MoA was classified based on the National Cancer Institute Drug Dictionary to ensure consistency, the categories were broad and may not fully distinguish clinically relevant therapeutic classes, such as immunotherapies versus non-immunotherapies or targeted versus non-targeted therapies. In addition, some categories may have included biologically and clinically heterogeneous agents with different molecular targets and development strategies. Therefore, this classification may have diluted potential associations between specific mechanisms of action and indication expansion, and findings related to MoA should be interpreted cautiously.

The data were extracted from the earliest available Phase 1 and 3 clinical trials, which may not necessarily correspond to the indications for which approval was ultimately granted. In addition, sponsors did not publicly report clinical trial results for some drugs, which could introduce selection bias. Furthermore, we did not comprehensively evaluate clinical efficacy across other tumor types. Thus, we could not determine whether drugs with only a single indication failed to obtain additional indications because of insufficient clinical efficacy in other tumor types. In particular, limited clinical activity outside the initially approved indication may be an important reason why some drugs remain approved for only a single indication.

Some Phase 1 and Phase 3 clinical efficacy data were unavailable or were excluded when the corresponding studies were conducted in hematologic malignancies or when efficacy was assessed using endpoints other than ORR and/or PFS, such as in studies of prostate cancer. Because this study was designed as a descriptive and exploratory analysis with a limited sample size, formal sensitivity analyses or missing-data analyses were not performed. Therefore, the findings may be subject to selection and reporting biases, and caution is warranted when interpreting results based on clinical efficacy data.

Company size was classified using annual revenue as a pragmatic surrogate for financial capacity. However, annual revenue alone may not fully reflect a company’s ability to pursue multi-indication development. In addition, this analysis did not account for partnerships, licensing arrangements, or co-development agreements, which may substantially influence the ability to conduct multiple indication trials.

## 4. Conclusions and Perspectives

Our narrative and qualitative analysis suggests that tumor type and clinical efficacy at the time of initial approval may be related to the subsequent acquisition of multiple indications, whereas clear patterns were not identified for other factors. Only 1 of the 9 drugs initially approved for rare cancers obtained additional indications, compared with 27 of 39 drugs first approved for non-rare cancers.

With respect to efficacy, agents demonstrating an ORR ≥ 40% or PFS ≥ 10 months were likely to gain additional indications. In contrast, no clear patterns were observed between the likelihood of indication expansion and either MoA or company size. Although potential associations were suggested between FDA special designation and number of indications, these findings should be interpreted cautiously since no formal statistical testing was performed.

Multiple strategies exist for expanding indications [42,43], such as addition of new tumor types or broadening of treatment lines. Our findings suggest that several confounding factors underlie these processes. Nevertheless, tumor type and efficacy at the time of initial approval appeared to be consistently associated with subsequent indication expansion regardless of approach.

Expanding indications to reach a broad patient population and maximize therapeutic and commercial value is a key component of oncology drug lifecycle management. In addition to identifying potential factors related to of multi-indication approval, this study also demonstrates that even drugs with modest efficacy may reach specific patient populations through careful selection of target indications. Ultimately, precise identification of the most appropriate indication for each agent is critical. By characterizing the factors potentially related to indication expansion, this study provides exploratory insights that may inform corporate R&D strategy and evidence-based decision-making in oncological drug development. Future studies should conduct confirmatory multivariable quantitative analyses to evaluate whether the observed patterns remain after adjustment for relevant confounding factors, including follow-up time, selection and reporting bias. In addition, comprehensive validation using datasets from multiple regulatory authorities would strengthen the external validity and generalizability of these findings. Overall, this exploratory study provides a foundation for future confirmatory research by generating clinically and strategically relevant hypotheses regarding factors associated with indication expansion in oncology drug development.

## Figures and Tables

**Table 1:** Summary of characterization by number of approvals.

Category	Subcategory	Single Indication	Multi-Indications	Total
MoA	Inhibitor	12	15	27
	multi-kinase inhibitor	3	4	7
	Cytotoxic therapy	2	2	4
	monoclonal antibody	3	4	7
	Antibody-drug conjugate	0	3	3
Tumor for initial approval	Rare tumor	8	1	9
	Non-rare tumor	12	27	39
Phase 1 ORR	<5%	3	2	5
	≥5 and <20%	3	7	10
	≥20 and <40%	0	6	6
	≥40%	1	7	8
	Not reported	13	6	19
Phase 3 ORR	<5%	0	1	1
	≥5 and <20%	4	5	9
	≥20 and <40%	8	4	12
	≥40%	3	12	15
	Not reported	4	7	11
Phase 3 PFS	<4 months	3	5	7
	≥4 and <7 months	7	2	9
	≥7 and <10 months	3	4	7
	≥10 months	1	13	14
	Not reported	6	5	11
Company	Emerging biopharma	4	5	9
	Other pharma	7	9	16
	Large pharma	9	14	23

MoA: Mechanism of action, ORR: Overall response rate, PFS: Progression-free survival.

## Data Availability

The data presented in this study are available on request from the corresponding author.

## Supplementary Materials

The following supporting information can be downloaded at: https://www.mdpi.com/article/10.3390/curroncol33060339/s1, Figure S1: Data collection flow chart. Figure S2: Factors that were modified when expanding indications from initial approval. Figure S3: The relationship between the mechanisms of action (MoA) of approved drugs and their indications. Figure S4: The relationship between company size and the number of approved indications.
