# Supplementary material for: Identifying Factors Associated with the Acquisition of Multiple Indications for Anticancer Drugs
Source: Curr Oncol. 2026 Jun 6;33(6):339. doi: 10.3390/curroncol33060339 (PMC13297679; doi:10.3390/curroncol33060339)
Supplement: Supplementary file 1 [file curroncol-33-00339-s001.zip › curroncol-4259373-supplementary.pdf]

Supplementary information

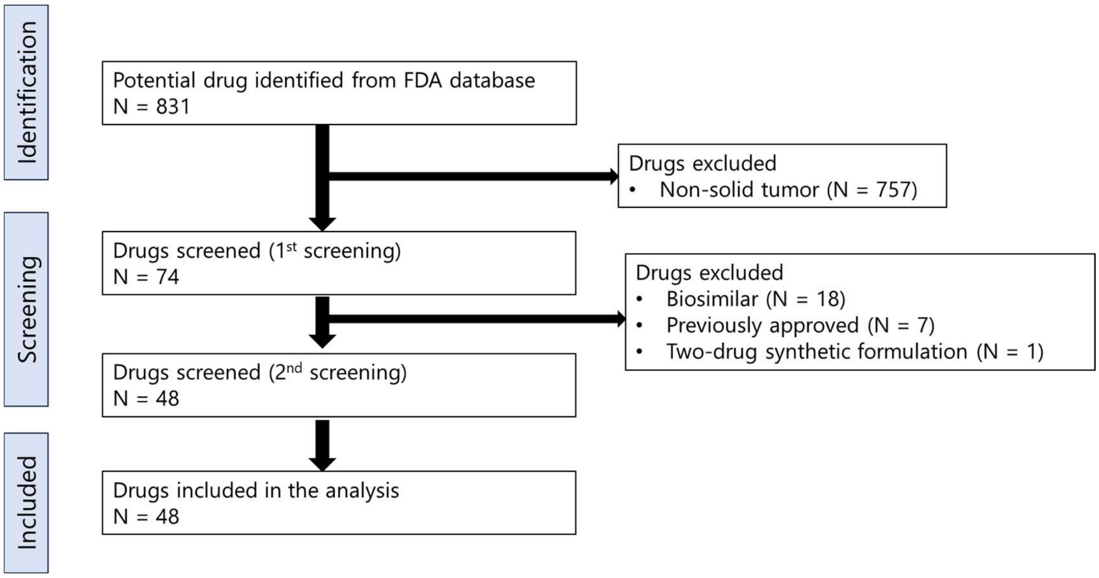

Figure S1. Data collection flow chart.

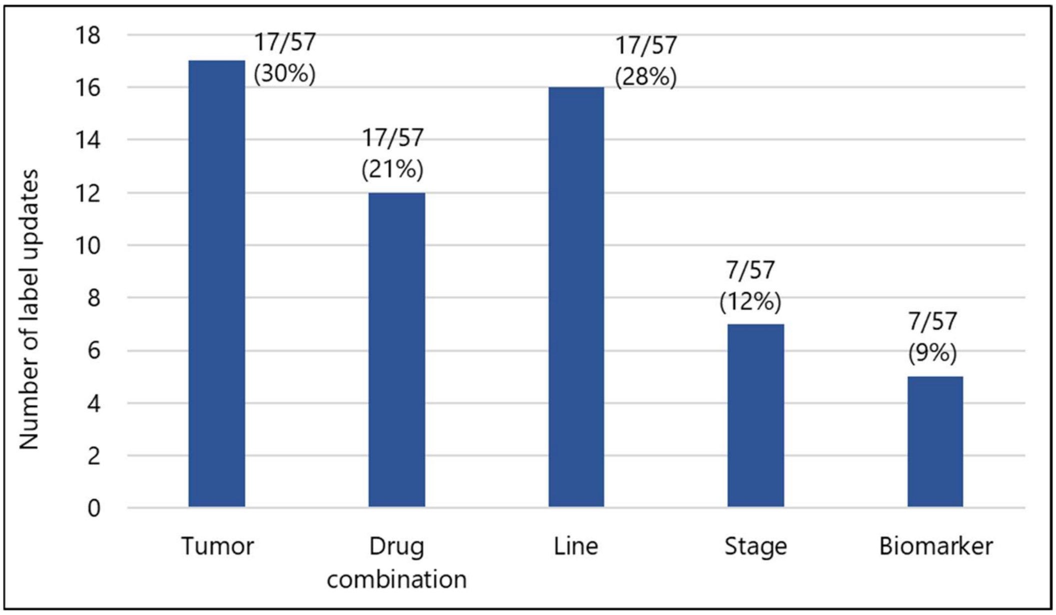

Figure S2. Factors that were modified when expanding indications from initial approval. Numbers above bars indicate n/N (%), where N represents the total number of label updates associated with indication expansion. Drug combination, line of therapy, disease stage, and biomarker were only evaluated when the cancer type remained unchanged.

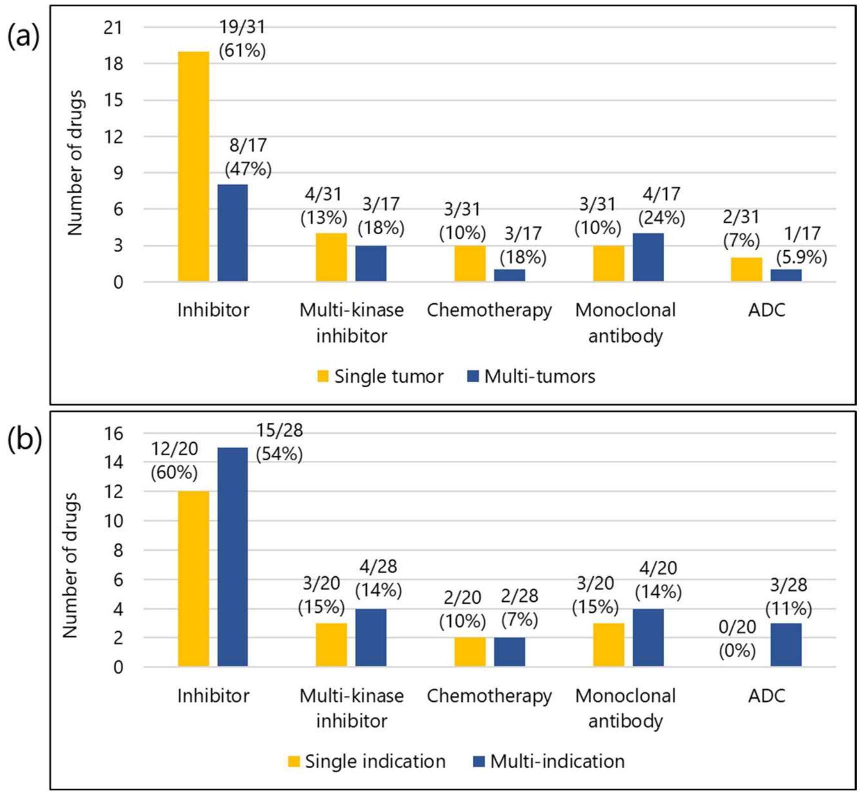

Figure S3. The relationship between the mechanisms of action (MoA) of approved drugs and their indications. Numbers indicate n/N (%). N represents the total number of drugs in each comparison group. (**a**) The numbers of drugs approved for single and multiple tumor types were compared for each MoA. (**b**) The numbers of drugs approved for single and multiple indications were compared for each MoA.

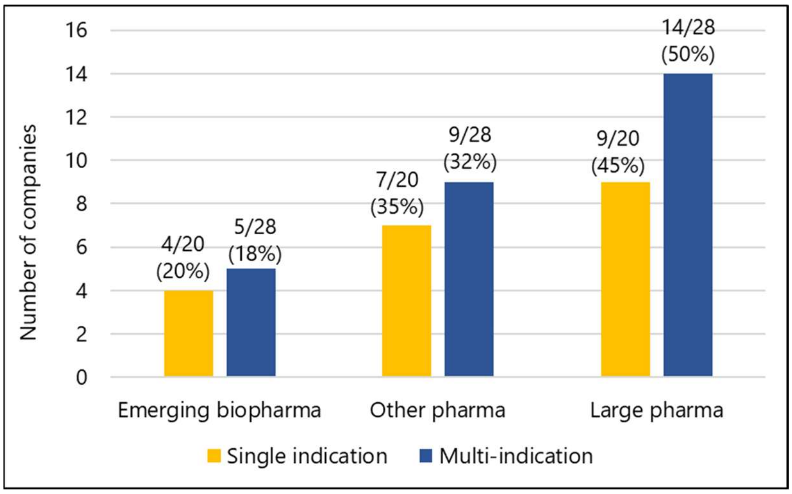

Figure S4. The relationship between company size and the number of approved indications. Numbers above bars indicate n/N (%). N represents the total number of drugs in each indication group
